# The cGAS-STING Pathway in Bacterial Infection and Bacterial Immunity

**DOI:** 10.3389/fimmu.2021.814709

**Published:** 2022-01-13

**Authors:** Nanxin Liu, Xiaoxiao Pang, Hua Zhang, Ping Ji

**Affiliations:** ^1^ Chongqing Key Laboratory of Oral Diseases and Biomedical Sciences, Chongqing Municipal Key Laboratory of Oral Biomedical Engineering of Higher Education, Stomatological Hospital of Chongqing Medical University, Chongqing, China; ^2^ Department of Obstetrics and Gynaecology, The First Affiliated Hospital of Chongqing Medical University, Chongqing, China

**Keywords:** cGAS, STING, bacteria, infection, innate immunity, CGAMP

## Abstract

Cyclic guanosine monophosphate (GMP)-adenosine monophosphate (AMP) (cGAMP) synthase (cGAS), along with the adaptor stimulator of interferon genes (STING), are crucial components of the innate immune system, and their study has become a research hotspot in recent years. Many biochemical and structural studies that have collectively elucidated the mechanism of activation of the cGAS-STING pathway with atomic resolution have provided insights into the roles of the cGAS-STING pathway in innate immunity and clues to the origin and evolution of the modern cGAS-STING signaling pathway. The cGAS-STING pathway has been identified to protect the host against viral infection. After detecting viral dsDNA, cGAS synthesizes a second messenger to activate STING, eliciting antiviral immune responses by promoting the expression of interferons (IFNs) and hundreds of IFN-stimulated genes (ISGs). Recently, the cGAS-STING pathway has also been found to be involved in response to bacterial infections, including bacterial pneumonia, melioidosis, tuberculosis, and sepsis. However, compared with its functions in viral infection, the cGAS-STING signaling pathway in bacterial infection is more complex and diverse since the protective and detrimental effects of type I IFN (IFN-I) on the host depend on the bacterial species and infection mode. Besides, STING activation can also affect infection prognosis through other mechanisms in different bacterial infections, independent of the IFN-I response. Interestingly, the core protein components of the mammalian cGAS-STING signaling pathway have been found in the bacterial defense system, suggesting that this widespread signaling pathway may have originated in bacteria. Here, we review recent findings related to the structures of major molecules involved in the cGAS-STING pathway and the effects of the cGAS-STING pathway in various bacterial infections and bacterial immunity, which may pave the way for the development of new antibacterial drugs that specifically kill bacteria without harmful effects on the host.

## Introduction

Bacterial infections caused by opportunistic pathogens or invading pathogenic bacteria are the major infectious diseases worldwide, causing many diseases, including pneumonia, periodontitis, tuberculosis, conjunctivitis, gastroenteritis, and sepsis (1–5). Although antibiotics have enabled great success in preventing and treating bacterial infections, overconsumption and misuse have unfortunately increased the prevalence of muti-drug-resistant (MDR) microbes (6, 7). A study that collected the total medical expenditure of inpatients in China from 2013 to 2015 reported an additional medical expenditure of US$15,557.25 per inpatient with a healthcare-associated infection (HAI) caused by antimicrobial resistance (AMR) infection compared with that without an HAI (8). The mortality rate of AMR infections increases every year, and it is estimated to kill 10 million people per annum by 2050 (9). Thus, the resurgence of bacterial infections has made them a pressing public health concern once again. In this context, researchers are striving to develop new strategies to treat bacterial infections and avoid drug resistance, among which immunotherapy is an important research direction due to its breakthroughs and advantages in the treatment of cancer and autoimmune diseases (7, 10).

**Figure 1:**
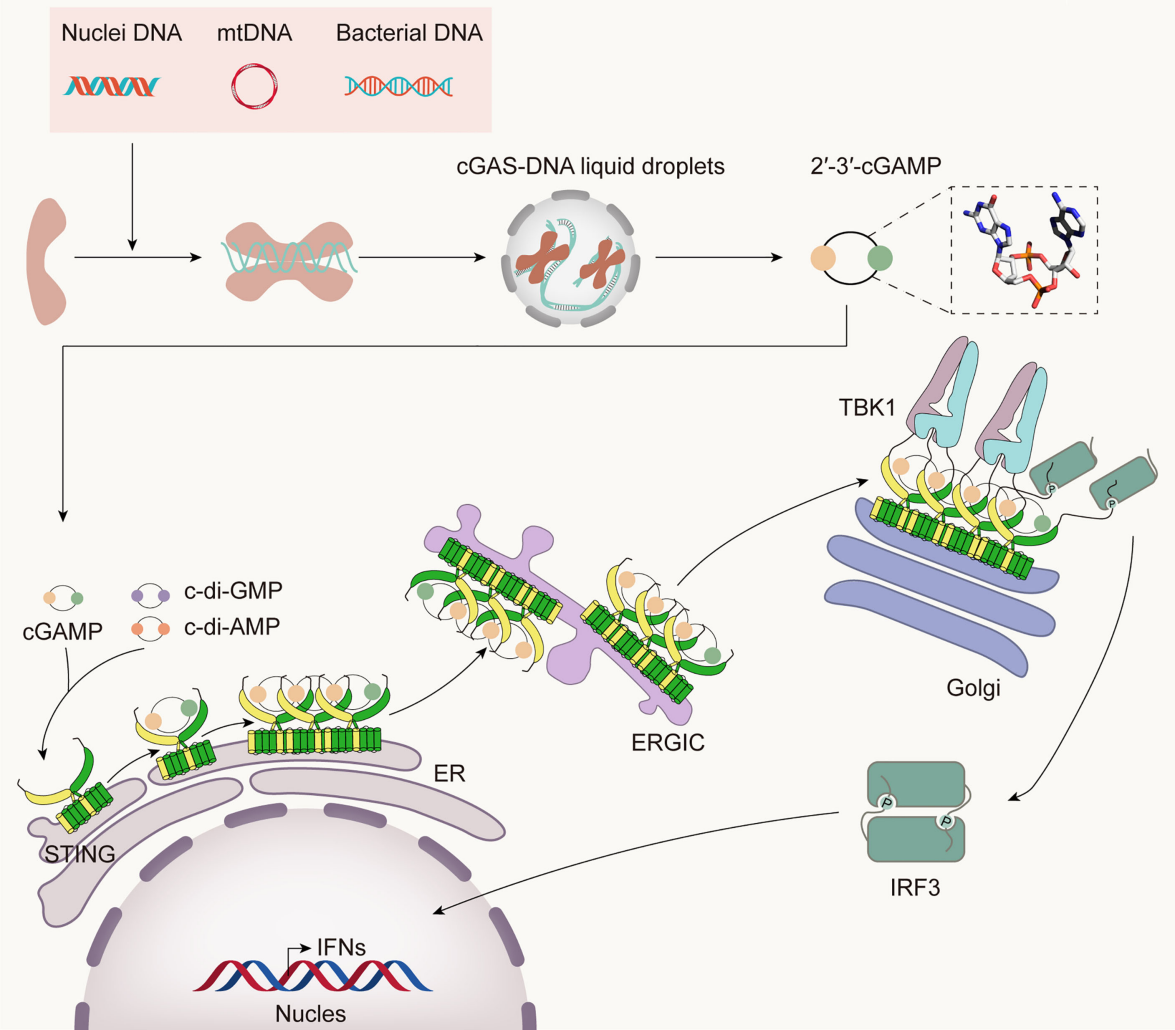
The cGAS-STING pathway. Cytosolic DNA from bacteria, damaged mitochondria, or nuclei is sensed by cGAS, leading to the formation of cGAS-DNA liquid droplets, in which ATP and GTP are catalyzed to 2’3’-cGAMP. It binds STING and initiates the traffic of STING from ER to Golgi and post-Golgi compartments. During the translocation process, STING recruits TBK1 and IRF3. TBK1 phosphorylates STING in its CTT, then phosphorylates IRF3, which then translocates into the nucleus, inducing the expression of IFN-I and many other inflammatory cytokines.

Through a complex network of biological processes, the immune system protects the body from diseases by recognizing and eliminating invading pathogens to sustain the organism’s homeostasis. The mammalian innate immune system has evolved various pattern recognition receptors (PRRs) to detect pathogens and damage-associated molecular patterns (PAMPs and DAMPs) to trigger the host’s defense rapidly (11). DNA sensors recognize pathogen DNA or misplaced host DNA to initiate innate immune responses and shape adaptive immunity (12–14). cGAS has been identified as the main DNA sensor that can generate the second messenger 2’3’-cGAMP upon detection of cytosolic DNA. Then, STING binds with 2’3’-cGAMP, and the complex is transferred from the endoplasmic reticulum (ER) to the Golgi complex, leading to the activation of TANK-binding kinase 1 (TBK1) and interferon regulatory factor 3 (IRF3) for IFN-I and inflammatory cytokine production (15) (**Figure 1**).

Over evolutionary time, cGAS has adopted multiple detection strategies to recognize various pathogens sensitively. First, cGAS is located in the cytoplasm, plasma membrane, and nucleus, allowing it to rapidly recognize DNA in different infectious contexts and initiate a downstream immune transcription cascade (16–19). Second, the sensitivity of cGAS to DNA can be increased by various factors during infection. For example, DNA that is prearranged by mitochondrial transcription factor A (TFAM) or modified by reactive oxygen species (ROS) can facilitate the detection of DNA by cGAS (20, 21). Third, inflammatory cytokines or cGAMP released from infected cells act as alarm signals, giving rise to the activation of the cGAS-STING pathway in bystander cells (22).

This strong surveillance pathway has attracted intense attention in the field of innate immunity and is widely believed to be effective in preventing viruses from entry, replication, or budding during infection (23–26). Additionally, the cGAS-STING pathway has also been involved in bacterial infections, but it does not always defend against bacteria, sometimes promoting their replication and survival (27–29). Moreover, unlike the case in viruses, bacterial lipopolysaccharide (LPS) and cyclic di-nucleotides (CDNs, including c-di-AMP, c-di-GMP, 2’2’-cGAMP, and 3’3’-cGAMP) can activate the cGAS-STING pathway, in addition to bacterial DNA (30–35). Both extracellular CDNs (eCDNs) and intracellular CDNs (iCDNs) can activate STING independently of cGAS to initiate the host immune response (**Figure 1**), while recent studies have found that cGAS facilitates sensing of eCDNs to activate innate immunity (36). After eCDNs pass through the lipid bilayer of cells *via* a folate-organic phosphate antiporter or clathrin-dependent endocytosis (37), they directly bind cGAS, induce dimerization, and promote the interaction between cGAS and STING. In this process, cGAS acts as a scaffold protein to nucleate perinuclear signalosomes containing eCDNs/cGAS/STING, thus activating STING in a 2’-3’-cGAMP-independent manner (36). LPS, another important PAMP, has recently been reported to induce the cytosolic release of mitochondrial DNA (mtDNA), which subsequently activates the cGAS-STING pathway (35). Interestingly, the cGAS-STING pathway, which mediates the development and prognosis of bacterial infection in mammalian cells in various ways, has been found to originate as a bacterial immune system that confers immunological protection against viral infection (38). Therefore, a better understanding of the role of the cGAS-STING pathway in bacterial infection and bacterial immunity will be of great value in many areas of research, such as the development of small molecule drugs targeting the bacterial cGAS-STING pathway without adverse effects on the host.

In this review, we summarize current knowledge on structural insights into the cGAS-STING signaling pathway and further review the activation mechanism and specific functions of the cGAS-STING pathway during the infection of intracellular gram-positive bacteria, including *Listeria monocytogenes* and *Staphylococcus aureus*; extracellular gram-positive bacteria including *Streptococcus pyogenes* and *Streptococcus pneumoniae*; intracellular gram-negative bacteria including *Brucella abortus*, *Burkholderia pseudomallei*, and *Francisella tularensis*; and extracellular gram-negative bacteria, such as *Pseudomonas aeruginosa*; and *Mycobacterium tuberculosis*, with a special focus on LPS due to its controversial role in the activation of the cGAS-STING pathway. Next, we present recent advances in understanding how the bacterial cGAS-STING pathway protects bacteria against phage infection and discuss the similarities and differences of the cGAS-STING pathway in bacteria and humans. The cGAS-STING pathway, which protects both bacteria and humans, has become a shining star in innate immunity. New ideas for treating bacterial infections may be discovered by reviewing the structure, signal transduction process, and role of the cGAS-STING pathway in bacterial infection and bacterial immunity.

## Structural Mechanism of the Activation of the cGAS-STING Pathway

In 2013, Chen’s team discovered the existence of cGAS in mammalian cells and its ability to synthesize cGAMP as the second messenger to activate STING directly (39, 40). The cGAS-STING pathway has since been identified to be extensively involved in various physiological and pathological processes (41). Due to its important role in immunity, many studies have analyzed the structure of key proteins and molecules in this pathway. Early structural studies of inactive human cGAS (hcGAS), mouse cGAS, and other mammalian homologs did not provide structural information of DNA recognition by activated hcGAS (42–46). Recently, researchers have identified the structural mechanism of dsDNA sensing by hcGAS in an active conformation (47) and cGAS inhibition by nucleosomes (48, 49), deepening our understanding of cGAS activation and providing guidance for the design of drugs that target cGAS. Since STING was discovered in 2008, a large number of studies have carefully analyzed the structure of STING, generating an elegant model of STING activation in which upon cGAMP binding, STING rotates inwardly toward the ligand-binding pocket, closes its ligand-binding pocket, and releases its CTT to recruit TBK1 and IRF3 (50–52). Here, we review recent high-impact structural work on cGAS and STING to increase our understanding of this signaling pathway at atomic resolution and promote the development of novel therapeutics for cGAS-STING-related diseases.

## cGAS

**Figure 2:**
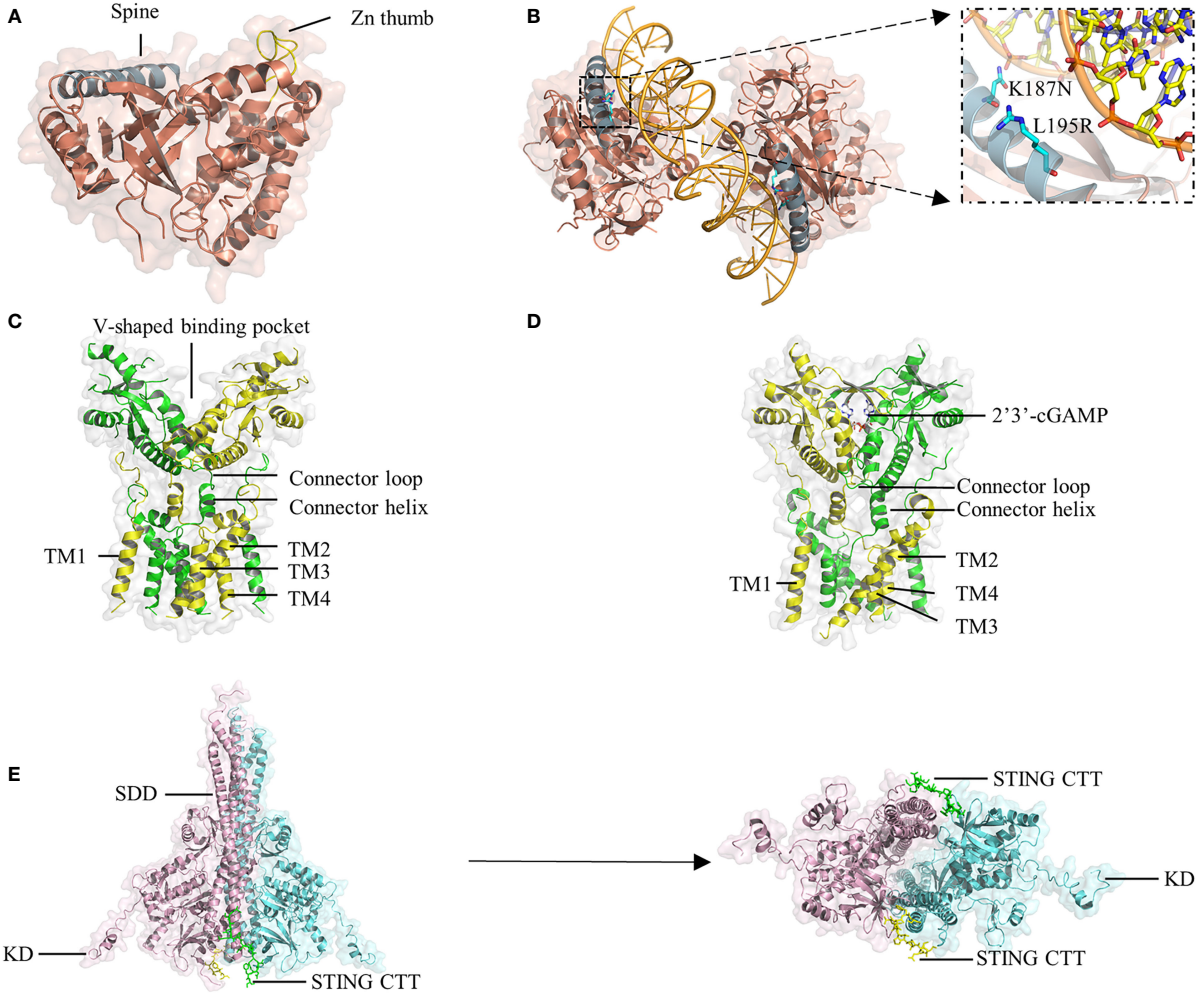
Crystal structure of cGAS, STING, and TBK1. **(A)** The hcGAS model is shown as ribbon representation with annotated structures including ‘spine’ in blue and ‘Zn thumb’ in yellow. **(B)** Schematic and overview of the 2:2 hcGAS:dsDNA complex structure. Zoom-in cutaways of the locations of K187 and L195 substitutions in hcGAS that are responsible for its long dsDNA preference. **(C)** Representation of the structure of full-length human STING in the apo state. **(D)** Structure of STING bound to cGAMP. **(E)**, Ribbon representations of the structure of mouse TBK1 in complex with the human STING CTT in a side view (left) and a bottom view (right).

cGAS belongs to the structurally conserved cGAS/DncV-like nucleotidyltransferase (CD-NTase) superfamily, consisting of an N-terminal domain and a C-terminal catalytic domain. The catalytic domain comprises the NTase core and Mab21 domains and adopts a bilobed structure that comprises a central catalytic domain and two different positively charged surfaces (41, 53). A long helix ‘spine’ at the N-terminus of the catalytic domain bridges the N-terminal lobe, which possesses the NTase fold with a two-leaved, highly twisted β-sheet, and the C-terminal lobe, which contains a tight helix bundle (**Figure 2A**) (54, 55). The catalytic site is located at the edge of the deep groove between the two lobes of cGAS. Once the positively charged surface of cGAS interacts with dsDNA in a sequence-independent manner, a significant structural switch occurs such that the catalytic pocket of cGAS is rearranged to initiate the cyclization of guanosine triphosphate (GTP) and adenosine triphosphate (ATP).

A unique protrusion called the ‘Zn thumb’ connects the two lobes and promotes the interaction of cGAS and the sugar-phosphate backbone of the DNA] duplex (**Figure 2A**) (45). Binding to dsDNA brings the two lobes of cGAS close to each other and activates cGAS (44, 46, 56). Four cGAS molecules assemble into a 2:2 mouse cGAS-dsDNA complex after binding dsDNA through extensive electrostatic interactions and hydrogen bonds. In these complexes, two dsDNA molecules are cross-linked with a cGAS dimer, and the two cGAS dimers bind to the dsDNA asymmetrically to avoid steric clashes of the two dsDNA duplexes (45). Similar to the mouse model of the cGAS-dsDNA complex, cGAS can oligomerize to a functionally active 2:2 cGAS-dsDNA state (41). Although oligomerization is beneficial for regulating enzymatic function, it is unclear whether it is necessary for cGAS activation.

HcGAS can discriminate the length of DNA and prefers long dsDNA, causing its cGAMP production level to be significantly lower than that of other mammals. Both the human-specific substitutions K187N and L195R (47) (**Figure 2B**) and the ladder-like networks formed between cGAS dimers and dsDNA (20) may explain the preference of human cGAS for long dsDNA, leading to a reduced immune response by reshaping the balance between the sensitivity and tolerance of dsDNA. In cGAS-DNA liquid droplets, a linear dinucleotide 5’-pppG(2’-5’)pA is first formed and then flips over to promote the generation of the second 3’-5’ phosphodiester bond, resulting in the formation of 2’3’-cGAMP (39, 57). Moreover, in addition to a positively charged surface, another cGAS-dsDNA interface (labeled site-C) was found to enhance the enzymatic activity of cGAS. These studies will provide insights facilitating the design of drugs targeting the active site of cGAS.

## STING

As an adaptor protein, STING is the core component in the signaling cascade of the innate immune response (58, 59). The small protein (~40 kDa) is anchored to the ER membrane by an N-terminal portion containing four transmembrane helices (TM1-4). The C-terminal domain (CTD) of STING consists of a ligand-binding domain (LBD) that is responsible for binding 2’3’-cGAMP and CDNs, and a C-terminal tail (CTT) that is capable of binding TBK1, both of which face the cytosol (60, 61). Recently, cryo-electron microscopy analyses have revealed that STING in apo states exists as a dimer in which a domain-swapped architecture is adopted through organizing the eight transmembrane helices and two CTDs (52). In the dimeric TM domain, one layer is the central layer formed by TM2 and TM4, and the other layer is the outer layer composed of TM1 and TM3. The LBD domain contains five β strands and four α helices, of which α-helix 1 is linked to TM4 by the connector helix (residues 141-149) and connector loop (residues 150-156) (52). Two STING CTDs dimerize mainly *via* hydrophobic interactions, generating a V-shaped binding pocket (**Figure 2C**).

Upon cGAMP binding, a four-stranded β-sheet lid-like structure forms over the ligand-binding pocket of STING to hold cGAMP tightly; this is described as the ‘closed’ conformation (**Figure 2D**). Interestingly, the crystal structure of STING bound to c-di-GMP is in an ‘open’ conformation, similar to the structure of apo STING (59, 62, 63). Moreover, a linked amidobenzimidazole compound can bind to and activate STING in an ‘open’ structure (64). However, the binding affinity of human STING to c-di-GMP is weaker than that of STING to cGAMP (65). Therefore, it is likely that closure of the ligand-binding domain is not required for STING activation but is beneficial for increasing binding affinity (50). Another conformational change induced by cGAMP is that the LBD of STING rotates 180° relative to the TM domain, causing the two connector LBDα1 elements, which form a right-handed crossover in the apo state, to become parallel to each other (52).

Moreover, cGAMP initiates intracellular transport of STING by interrupting the interaction between STING and ER-resident protein stromal interaction molecule 1. Then, STING travels anterogradely from the ER to the Golgi apparatus depending on SEC24C, a component of the canonical coat protein complex II (COPII), *via* the ER-Golgi intermediate compartments (ERGICs) upon STING oligomer formation (66). STING dimers are not the only functionally active units in STING; tetramers and higher-order oligomers are also observed to form through the rotation of the LBD and the conformational change of the connector loop (41). The polymerization of STING also contributes to its activation (67–69), but the size and minimum activation length of functional STING polymers are unclear.

## The STING CTT and TBK1

The STING CTT plays a vital role in activating STING-TBK1-IRF3 by mediating the recruitment and activation of TBK1 and IRF3. TBK1 exits as an elongated dimer containing the scaffold and dimerization domain (SDD), a ubiquitin-like domain (ULD), and the kinase domain (KD) (**Figure 2E**) (51, 70). The N-terminal lobe of the KD interacts with the SDD of the dimer partner. The TBK1-binding motif (TBM) within the STING CTT interacts with TBK1 at many sites, including a groove between the KD of one monomer and the SDD of another monomer in the same TBK1 dimer; a deep hydrophobic pocket in the middle of the groove; Phe585, Tyr55, and Arg405 in the SDD; and Lys 30 in the KD (51). Although the TBK1 dimer binds flexibly to C-terminal TBMs from the STING dimer, it has little or no contact with the LBD of STING. Recently, some residues in the TBM were identified to constitute a highly conserved PLPLRT/SD motif, which is responsible for TBK1 recruitment (70). However, even if a large amount of TBK1 already binds to these preformed STING dimers on the ER, phosphorylation in *trans* will be blocked by steric hindrance. TBK1 can only be activated if parallel-stacking of STING homodimers is disrupted by the conformational change induced by cGAMP to interfere with CTT sequestration, which is essential for controlling basal STING activation (71). The TBK1 dimer binds two peptides from STING, and each peptide simultaneously binds two TBK1 monomers to form a 2:2 complex. In the STING-TBK1 complex, the autophosphorylation of TBK1 is mediated by the proximity in trans induced by adjacent STING molecules. High-order oligomerization of STING and TBK1 promotes the TNK1-induced phosphorylation of the serine residue in the pLxlS motif of STING, which not only enhances the binding of STING with TBK1 (51) but also provides a docking site for STING to bind IRF3. The recruited IRF3 is phosphorylated by adjacent TBK1 binding to the CTT of STING and then dimerizes and enters the nucleus to initiate the production of inflammatory cytokines, including IFN-I (**Figure 1**), proving that STING may scaffold the interaction between TBK1 and IRF3. Nuclear factor (NF)-κB signaling is also downstream of STING activation (72). However, it is unclear which of the two signaling pathways is activated or comes first. Moreover, although the important role of CTT in STING signal propagation has been confirmed, the detailed structure of CTT is still unknown.

## cGAS-STING Signaling Pathway in Bacterial Infection

**Figure 3:**
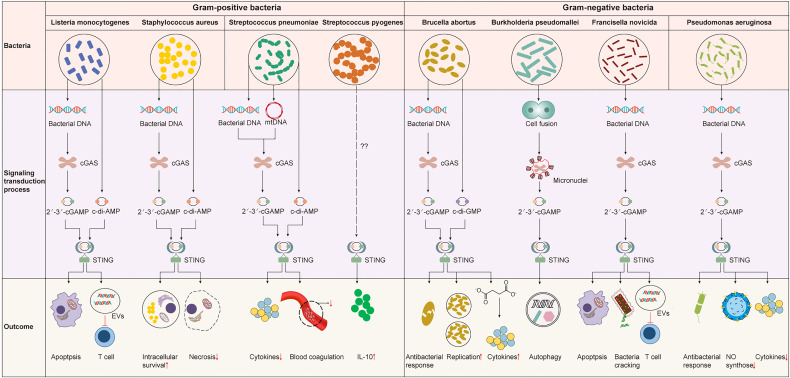
The cGAS-STING pathway in bacterial infection. The cGAS-STING pathway is widely involved in various bacterial infections. However, the signaling transduction process and outcome upon the activation of the cGAS-STING pathway are different. STING is activated by 2’3’-cGAMP produced by cGAS or bacterial CDNs to induce an IFN-I response that may be protective or detrimental for the host. STING can also influence the infection outcome by mediating other important biological or physiological processes like blood coagulation or metabolism independent of IFN-I response.

The immune system is required for protecting the body by controlling bacterial infections but can also lead to pathology. On the one hand, IFN-I induced by the cGAS-STING pathway can fight against bacterial infections and inhibit the overactivation of the immune response (73, 74). On the other hand, it had been identified to increase susceptibility to several bacteria, such as *Listeria monocytogenes* (75, 76). Furthermore, in the face of bacterial infection, the activation of STING not only initiates the IFN-I response but also interacts with bacteria by regulating metabolism or other downstream pathways (27, 77). Recently, c-di-AMP produced by live gram-positive bacteria, whether intracellular bacteria such as *Staphylococcus aureus* and *Listeria monocytogenes* or extracellular bacteria such as *Streptococcus pyogenes*, has been reported to induce a previously unappreciated cell-autonomous response by STING to elicit an augmented IFN-I response in host defense against infection caused by live bacteria (78). The mRNA of live gram-negative bacteria, rather than c-di-AMP, induces innate responses *via* STING-independent signaling pathways (79). Therefore, the activation mechanisms and effects of the cGAS-STING pathway differ between invading bacteria and infected tissues or cells. The interaction between the cGAS-STING pathway and gram-positive or gram-negative bacteria will be discussed in detail below (**Table 1** and **Figure 3**).

**Table 1:** The activation of the cGAS-STING pathway in various bacterial infections.

Pathogens	DNA	CDNs	IFN-I response induced by the cGAS-STING pathway	Additional functions of STING activation	Refs
*Staphylococcus aureus*	live *S. aureus* DNA	c-di-AMP	i. Promote macrophage polarization to an anti-inflammatory phenotype ii. Promote bacterial growth	Control pulmonary *S. aureus* infection	(80–89)
*Listeria monocytogenes*	*Listeria* DNA	c-di-AMP	Detrimental effect	i. Reduce the influx of inflammatory monocytes ii. Promote Evs formation, and dampen the immunological activity of T cells	(73, 76, 90–96)
*Streptococcus pneumoniae*	Pneumococcal DNA, mtDNA	c-di-AMP	Suppress inflammation-related damage and lethality	Regulate blood coagulation	(74, 77, 97–100)
*Streptococcus pyogenes*	Unclear	Unclear	Suppress the inflammatory response	Unclear	(101–103)
*Brucella abortus*	*B.abortus* DNA	c-di-GMP	i. Anti-bacterial response ii. Induce UPR expression to promote *B.abortus* replication	Mediate cellular metabolism to control *B.abortus* replication	(27, 28, 104–106)
*Burkholderia*	Micronuclei (dsDNA)	Unclear	Irrelevant to the antibacterial response	Autophagic cell death	(107–114)
*Francisella novicida*	*Francisella* DNA	Unclear	i. Induce cell apoptosis ii. Promote bacteriolysis and additional DNA release	Promote Evs formation, and dampen the immunological activity of T cells	(115–118)
*Pseudomonas aeruginosa*	*Pseudomonas* DNA	Unclear	Fight against bacterial infection	i. Enhance NO synthase expression ii. Inhibit proinflammatory cytokines	(119–122)
*Mycobacterium tuberculosis*	Mycobacterial DNA	c-di-AMP	Antibacterial defense	unclear	(1, 123–134)

## Intracellular Gram-Positive Bacteria

### Staphylococcus aureus

*Staphylococcus aureus* is the etiological pathogen of many diseases, ranging from superficial skin infections to life-threatening infections such as pneumonia (80, 81). The c-di-AMP released from *S. aureus* biofilms activates STING to upregulate the expression of IFN-I in macrophages. The STING-dependent IFN-I response promotes macrophage polarization to an anti-inflammatory phenotype, resulting in impaired *S. aureus* clearance and exacerbated infectious outcomes (82). Moreover, the cGAS-STING pathway can be activated in response to live but not dead *S. aureus* by sensing *S. aureus* DNA. During the early stage of *S. aureus* infection, the Toll-like receptor (TLR) and cGAS-STING pathways are both activated by live *S. aureus* but exhibit opposite roles in the host immune response to *S. aureus.* TLR signaling restricts infection, while the cGAS-STING pathway enhances bacterial growth (83, 84). The transcription and expression of IFN-I are well-known to be induced by phosphorylation of IRF3. However, IRF3 phosphorylation in dendritic cells acts independently of activating the cGAS-STING pathway to drive IFN-I expression in response to inactivated *S. aureus* treated by ultraviolet irradiation (85).

Meanwhile, the production of many cytokines induced by live or inactivated *S. aureus* is different (85, 86), suggesting that the viability of *S. aureus* may influence the host immune response to its infection. Previous studies reported that the cGAS-STING pathway was involved in initiating necroptosis in macrophages in an IFN-I-dependent manner (87, 88). However, in the context of *S. aureus* pneumonia, STING was reported to facilitate the restriction of *S. aureus* infection and protect the architecture and function of the lung by suppressing macrophage necroptosis (89). According to these studies, IFN-I induced by the cGAS-STING pathway seems to be related to immune evasion of *S. aureus*, while the activation of STING may also contribute to controlling pulmonary *S. aureus* infection. Therefore, the protective effect of STING against *S. aureus* pneumonia needs to be confirmed first. Next, how STING suppresses necroptosis in the presence of increased IFN-I needs to be further studied.

### Listeria monocytogenes

*Listeria monocytogenes* can cause listeriosis, which usually manifests as self-limited gastroenteritis, although a few cases may develop into sepsis, meningitis, and monocytosis, mainly in newborns, the elderly, and immunocompromised individuals (90, 91). The IFN-I response has been reported to stimulate excessive immune activation as an important component of listeriosis pathology (76, 92, 93). During *L. monocytogenes* infection, STING is activated by bacterial DNA-activated cGAS or another DNA sensor, IFI16, or by binding c-di-AMP secreted by *L. monocytogenes via* multidrug efflux pumps (MDRs), in both cases leading to the production of IFN-I (73, 94). Therefore, *L. monocytogenes* trigger IFN responses *via* the cGAS-STING pathway, making it a novel therapeutic target for listeriosis. Recently, the endogenous anaphylatoxins C5a and C3a have been found to suppress the expression of STING and phosphorylated TBK1 and IFN-β during *L. monocytogenes* infection, consequently attenuating the detrimental effect of IFN-β production, for example, inhibiting *L. monocytogenes-*mediated-apoptosis of immune cells (73). However, the STING-dependent immune response was suggested to defend against *L. monocytogenes* by reducing the influx of inflammatory monocytes and systemic bacterial loads during enterocolitis (95). This result indicated that in addition to the IFN-I response, STING activation in enterocolitis could also exert immunomodulatory effects through other mechanisms that need further investigation. *L. monocytogenes* has evolved to manipulate STING strategically for its benefit instead of evading it. For example, its DNA is sorted into extracellular vesicles (EVs) in a process that depends on STING, TBK1, and multivesicular body protein (MVB12b) and is then delivered to bystander cells to further activate the cGAS-STING pathway. Both EVs that dampened the immunological activity of T cells and increased IFN-I production in bystander cells promote the establishment of infection in a STING-dependent manner (96). Other intracellular bacteria, including *Francisella tularensis* and *Legionella pneumophila*, also adopt this strategy to impair antibacterial defense. According to these studies, the cGAS-STING pathway dominantly enhances host susceptibility to *L. monocytogenes* by upregulating IFN-I production but may play an antibacterial role in some diseases in an IFN-independent manner. However, the underlying mechanism of STING resistance to *L. monocytogenes* infection remains unclear.

## Extracellular Gram-Positive Bacteria

### Streptococcus pneumoniae

*Streptococcus pneumoniae* can colonize the upper respiratory tract asymptomatically or cause diseases such as bacterial pneumonia and meningitis (97, 98). It enters mature phagolysosomes and releases nucleic acids and c-di-AMP into the host cytosol *via* a secretion system or following partial lysis. *S. pneumoniae* c-di-AMP initiates host innate immune responses by activating STING directly and increasing the expression of IFN-β (99). IFN-β production is also induced by pneumolysin (Ply), a pore-forming protein and a major virulence factor of *S. pneumoniae.* It can initiate oxidative damage to mitochondria in macrophages, leading to the cytosolic translocation of mtDNA and triggering IFN-β expression in a cGAS-STING dependent manner (97). Although cGAS has been reported to sense pneumococcal DNA and then stimulate STING, resulting in the activation of IFN-I response in mouse macrophages, how pneumococcal DNA enters the cytoplasm of host cells has not been clarified. Notably, IFN-I induced by *S. pneumoniae* has been identified to be protective to the host by suppressing inflammation-related damage and lethality (74). Moreover, STING can regulate coagulation by increasing cytosolic calcium and then triggering the release of a key initiator of blood coagulation independent of TBK1 or IRF3 activation, thus limiting the severity of sepsis caused by *S. pneumoniae* (77). These results reveal that the cGAS-STING signaling pathway plays an immunoprotective role in *S. pneumoniae* infection by generating IFN-I and regulating coagulation. Conversely, *Ruiz-Moreno* et al. reported that although the cGAS-STING pathway sensed *S. pneumoniae* invasion and induced an IFN-I response, it was dispensable for defense against *S. pneumoniae* infection (100). Hence, the exact role of the cGAS-STING pathway in anti-pneumococcal defense needs to be further studied.

### Streptococcus pyogenes

*Streptococcus pyogenes* can cause a range of symptoms, from mild illness to life-threatening infections such as necrotizing fasciitis (101). A previous study suggested that the activation of the STING pathway and the subsequent increase in IFN-I production were probably initiated by cGAS upon sensing cytosolic nuclear acids in *S. pyogenes*-infected macrophages and dendritic cells (102). However, *Movert* et al. reported that *S. pyogenes* activated the STING pathway independent of cytosolic dsDNA because neither bacterial nor host DNA could be detected in the cytosol of *S. pyogenes* infected macrophages (103). In addition, *S. pyogenes* promoted the production of anti-inflammatory IL-10 to curb the inflammatory response through the activation of the STING signaling pathway mediated by the streptococcal M protein, a surface-anchored virulence factor (103). Thus, IFN-I signaling was exploited by *S. pyogenes* to avoid life-threatening inflammation. Although the activation of STING has been suggested to be independent of cGAMP generated by cGAS during *S. pyogenes* infection (103), whether it is activated by c-di-AMP synthesized by *S. pyogenes* remains unknown.

## Intracellular Gram-Negative Bacteria

### Brucella abortus

*Brucella abortus* replicates and survives in macrophages and dendritic cells by forming *Brucella*-containing vacuoles (BCVs) (104). *B. abortus* c-di-GMP binds to STING, triggers the TBK1-IRF3 signaling cascade, and initiates the IFN-I response independently of cGAS. Subsequently, IFN-I signaling upregulates the expression of guanylate-binding proteins (GBPs), which can promote *B. abortus* DNA translocation from the BCV to the cytosol to activate AIM2 and increase IL-1β secretion (105). However, whether cGAS senses *B. abortus* genomic DNA and further amplifies the IFN-I signaling pathway and whether cGAS and AIM2 can cooperate to protect the host from *B. abortus* infection remain unclear. Moreover, STING controls *B. abortus* replication by regulating the metabolic reprogramming of macrophages. STING increases the levels of succinate to stabilize hypoxia-inducible factor-1 alpha (HIF-1α), resulting in the production of proinflammatory cytokines to limit *B. abortus* infection (27). However, the STING and IFN-I signaling pathway was reported to be required for an increase in unfolded protein response (UPR) expression, which facilitates *B. abortus* replication (28). According to these studies, STING is critical in host protective immunity by triggering the IFN-I pathway and mediating cellular metabolism. Thus, we speculate that when STING responds to *B. abortus* infection, the response that defends against *B. abortus* may be stronger than that promoting bacterial replication. The specific mechanism of these antagonistic effects needs to be further elucidated. In addition, *B. abortus* can decrease the expression of STING in the early stage of infection by upregulating miR-24-2 *via* a type IV secretion system, providing an environment in which *Brucella* can replicate in host cells free from the threat of the cytosolic surveillance pathway (106). In the future, appropriate utilization of miRNAs targeting STING may prevent excessive inflammation induced by *B. abortus* infection.

### Burkholderia

*Burkholderia pseudomallei* affects immune function *via* manipulating the caspase system, causing melioidosis with high morbidity and mortality (107, 108). Unlike many other intracellular bacteria, *B. pseudomallei* induces host cell fusion, an important aspect of melioidosis pathogenesis (109, 110). Cell fusion leads to the formation of multinucleated giant cells (MNGCs), which facilitate intracellular dissemination of *B. pseudomallei* without exposure to extracellular host defenses or antimicrobials (111, 112). cGAS responds to *B. pseudomallei* infection by colocalizing with micronuclei formed during aberrant and abortive mitotic events (110). Upon activation of the cGAS-STING pathway, autophagic cell death is induced to limit aberrant cell division and cellular transformation. Intriguingly, transcriptional changes rather than IFN-I production are induced by the cGAS-STING pathway (110). *Burkholderia thailandensis*, a close relative of *B. pseudomallei* (113), also invades the cytosol, induces cell-cell fusion allowing intracellular diffusion, and forms MNGCs to promote bacterial replication. Downstream signaling molecules rather than upstream molecules, such as cGAS/STING for IFN-I, are critical to restrict MNGC formation and bacterial actin motility during *B. thailandensis* infection (114). These results indicate that the IFN-I production induced by the cGAS-STING pathway may be irrelevant to the antibacterial response.

### Francisella novicida

During *Francisella novicida* infection, cGAS and interferon-γ inducible protein 16 (IFI16) synergistically sense cytosolic *Francisella* DNA to fully activate the STING-dependent IFN-I response, which plays a detrimental role by inducing apoptotic cell death (115, 116). In addition, IFN-I signaling is required to activate the AIM2 inflammasome by promoting bacteriolysis and bacterial DNA release (117). In turn, the AIM2 inflammasome negatively regulates the IFN-I response by mediating the interferon regulatory module, thus restraining the cGAS-driven IFN-I response (118). However, IFN-I signaling overrides the protective AIM2 inflammasome responses and plays a dominant role in exacerbating *F. novicida* infection (117). Since the relative expression levels of cGAS, IFI16, and AIM2-like receptors vary in different cell types, the specific reaction and interaction mechanisms in specific cells need to be further studied. Furthermore, *F. novicida* can suppress antibacterial defense by forming EVs in a STING-dependent manner to promote infection, similar to *L. monocytogenes.*

## Extracellular Gram-Negative Bacteria

### Pseudomonas aeruginosa

*Pseudomonas aeruginosa* is an opportunistic bacterium that can easily cause infection in immunocompromised people (119). The cGAS-STING pathway has been reported to have a protective effect in *P. aeruginosa* infection by promoting host resistance against *P. aeruginosa* infection and negatively modulating host inflammatory responses (120, 121). After penetrating *P. aeruginosa* into host cells, its genomic DNA can be sensed by cGAS, which mediates the IFN-I response by activating STING. Although the secretion of IFN-I increased, the level of proinflammatory cytokines decreased due to the inhibition of p38, JNK, ERK, and NF-κB activity by STING. In addition, STING can enhance NO synthase expression to eliminate invaded *P. aeruginosa* (121). Overall, cGAS-STING restrains P. aeruginosa infection by inducing the expression of IFN or NO synthase and protects host cells by suppressing excessive production of inflammatory cytokines. Recently, an X-ray-inactivated whole-cell vaccine was found to resist *P. aeruginosa* infection by stimulating the cGAS-STING pathway in dendritic cells (DCs) to foster its maturation which boosts T cells (122). Notably, the vaccine also protected against an MDR strain (122). Although the specific mechanism by which bacterial DNA enters the cytoplasm and whether host DNA is involved in the activation of cGAS during *P. aeruginosa* infection remains unclear, the critical role of the cGAS-STING pathway in the recognition and restriction of *P. aeruginosa* has been proven. In summary, immunotherapy targeting the cGAS-STING signaling pathway to treat AMR infections is a reliable line of research.

## Others

### Mycobacterium tuberculosis

*Mycobacterium tuberculosis*, neither gram-positive nor gram-negative bacteria, is the etiological agent of tuberculosis, which has been a major threat to human health since its discovery more than a century ago (1). *M. tuberculosis* survives primarily within macrophages, with the ESX-1 secretion system as the virulence factor. Numerous previous studies have suggested that the passive leakage of mycobacterial DNA into the cytosol with the help of ESX-1 can activate the cGAS-STING pathway to induce antibacterial defenses (123–127). What’s more, a recently revised model indicated that ESX-1 was involved in the mobilization of the cytosolic release of mitochondrial and nuclear host DNA by permeabilizing host membranes *via* genetically separable mechanisms (128). But the underlying mechanism in which ESX-1 affects the membrane integrity of the phagosome, nucleus, and mitochondria remains unclear. In addition, Mycobacterial c-di-AMP activates STING directly independent of ESX-1 (30, 129).

During the parallel evolution between host cells and *M. tuberculosis*, *M. tuberculosis* has developed many strategies to evade the surveillance of the cGAS-STING pathway to establish infection (130–133). For example, *M. tuberculosis* coding protein Rv0753c (MmsA) can not only decrease STING levels and subsequent IRF3 activation by binding and colocalizing with STING, but also facilitate STING autophagic degradation by binding with p62, resulting in the inhibition of STING-TBK1-IRF3 pathway (130); *M. tuberculosis* phosphodiesterase (PDE) can inhibit STING activation by cleaving both c-di-AMP and cGAMP to blunt the antibacterial response (133); *M. tuberculosis* isolates associated with severe tuberculosis can evade cGAS-STING surveillance system by accumulating mutations in the ESX components or generating sigA recognition boxes, and at the same time, IL-1β secretion caused by these isolates is significantly decreased (131). Furthermore, both CDNs-adjuvanted protein subunit vaccine and inhibitors of *M. tuberculosis* PDE can protect the host from infection by *M. tuberculosis* through eliciting a stronger anti-inflammatory response (132, 134). Thus, vaccine design and therapeutic development should consider genetic diversity and the continuous evolution of *M. tuberculosis* isolates in the future.

### LPS

LPS, localized in the outer layer of the membrane of gram-negative bacteria, has been widely recognized to cause various infections by activating TLR4. Extracellular LPS-induced activation of the TLR4-dependent cGAS-STING-NLRP3 axis is responsible for acute lung injury (ALI). LPS was reported to promote the cytosolic release of mtDNA, which activated cGAS to induce inflammation and oxidative stress in BMDMs. cGAS inhibition could mitigate inflammation by blocking the activation of STING and the NLRP3 inflammasome in LPS-treated BMDMs (135). In contrast, *Cao* reported that cGAS was dispensable for the LPS-induced inflammatory response in BMDMs because LPS and IFN-γ elicited a robust increase in inflammatory cytokines in both cGAS-null and wild-type cells (136). Both studies found that inflammatory cytokine secretion was significantly decreased in cGAS-silenced or cGAS-knockout BMDMs, while higher mRNA levels of these inflammatory cytokines were observed in cGAS-null cells than in cells not treated with LPS, which indicated that other critical factors participate in promoting the inflammatory response in LPS-stimulated BMDMs. The difference in roles of cGAS in the inflammatory response of BMDMs might also be explained by the different treatment modalities used, one stimulated by LPS only and the other stimulated by LPS in combination with IFN-γ. As for STING, it is activated by cGAMP produced by cGAS to aggravate inflammation and oxidative stress in LPS-induced ALI (135).

Meanwhile, its expression in BMDMs is upregulated by the transcription factor c-Myc, the expression of which is enhanced by LPS (135). However, LPS was reported to induce the perinuclear translocation of STING mediated by TLR4 to activate the phosphorylation and nuclear translocation of IRF3 in mice or neonatal rat cardiomyocytes (NRCMs) without affecting the protein expression of STING (137). Therefore, STING activation may not necessarily be accompanied by increased protein expression in some cell types. STING plays a vital role in LPS-induced cardiac dysfunction, inflammation, apoptosis, and pyroptosis by triggering the activation of the NLRP3 inflammasome in an IRF3-dependent manner (137). Above all, targeting the cGAS-STING pathway is an efficient strategy to inhibit LPS-induced acute lung injury and cardiac dysfunction.

Intriguingly, STING expression was repressed by LPS in human cells through a metabolism-dependent pathway (138). During the metabolic reprogramming of human cells after LPS stimulation, a cell-permeable derivative of itaconate (4-octyl-itaconate, 4-OI) accumulated and then activated the transcription factor Nrf2 (nuclear factor (erythroid-derived 2)-like 2), which decreased STING expression and negatively regulated the STING-dependent IFN-I response. However, inhibition of STING by Nrf2 following LPS treatment was observed in neither BMDMs nor RAW264.7 cells. Additionally, the phosphorylation and expression of STING were enhanced in LPS-treated mouse cells within 12 h, and the peak was reached at 6 h, whereas LPS inhibited the expression of STING in human cells from 24 to 72 h (138), and this inhibition strengthened with time. These results suggest that the effect of LPS on STING may be cell-dependent and time-dependent. Additionally, it is necessary to study further whether LPS can inhibit the expression of STING in mouse cells after prolonged treatment, whether the effect of LPS on STING is consistent in human and mouse macrophages, and what role STING plays in the crosstalk between metabolism and innate immunity; these findings will benefit the identification of a potential treatment target in STING-dependent inflammatory diseases.

LPS from extracellular bacteria can also gain access to the cytosol of host cells through endocytosis mediated by outer membrane vesicles (OMVs) in a TLR4-independent manner (139). Internalized LPS activates the noncanonical caspase-11 inflammasome, which cleaves Gasdermin D (GSDMD), leading to the generation of GSDMD N-terminal fragments (GSDMD-NT) and subsequent pyroptosis in bacterial sepsis (140, 141). Recently, activation of caspase-11 and formation of GSDMD-NT by cytosolic LPS were reported to initiate a decrease in mitochondrial membrane potential (MMP) and the release of mtDNA into the cytosol in lung microvascular endothelial cells (LMVECs). The DNA-sensing cGAS-STING pathway is activated by cytosolic mtDNA, leading to the impairment of endothelial regeneration after inflammatory lung injury (35). It is worth noting that internalized LPS induces a decrease in MMP, while extracellular LPS causes an increase in MMP (35, 142). Although this difference may be due to differences in cell type, the finding that intracellular LPS transfection activated cGAS-STING through the caspase-11-GSDMD pathway independent of TLR4 activation reveals that LPS can induce mitochondrial injury through different intracellular and extracellular pathways, which requires further investigation (**Table 2**).

**Table 2:** The cGAS-STING pathway response to LPS.

LPS administration	cGAS expression	Cytosolic mtDNA level	cGAMP production	STING expression	STING localization	STING phosphorylation	The role of the cGAS-STING pathway	Refs
LPS transfection	NA	upregulate	upregulate	NA	NA	NA	LPS activates the cGAS-STING pathway to promote inflammatory injury	(35)
LPS added to the culture medium	Upregulate	upregulate	upregulate	upregulate	NA	upregulate	LPS activates the cGAS-STING pathway to promote lung injury	(135)
LPS added to the culture medium	NA	NA	NA	NA	NA	NA	cGAS is dispensable for the inflammatory response induced by LPS	(136)
LPS added to the culture medium	NA	NA	NA	No change	Promote its perinuclear translocation	NA	LPS induces cardiac inflammation *via* the STING-IRF3-NLRP3 axis	(137)
LPS added to culture medium	NA	NA	NA	downregulate	NA	NA	LPS negatively regulate STING *via* an Nrf2-dependent way	(138)

NA, not available.

## cGAS-STING Signaling Pathway in Bacterial Immunity Against Viral Infection

**Figure 4:**
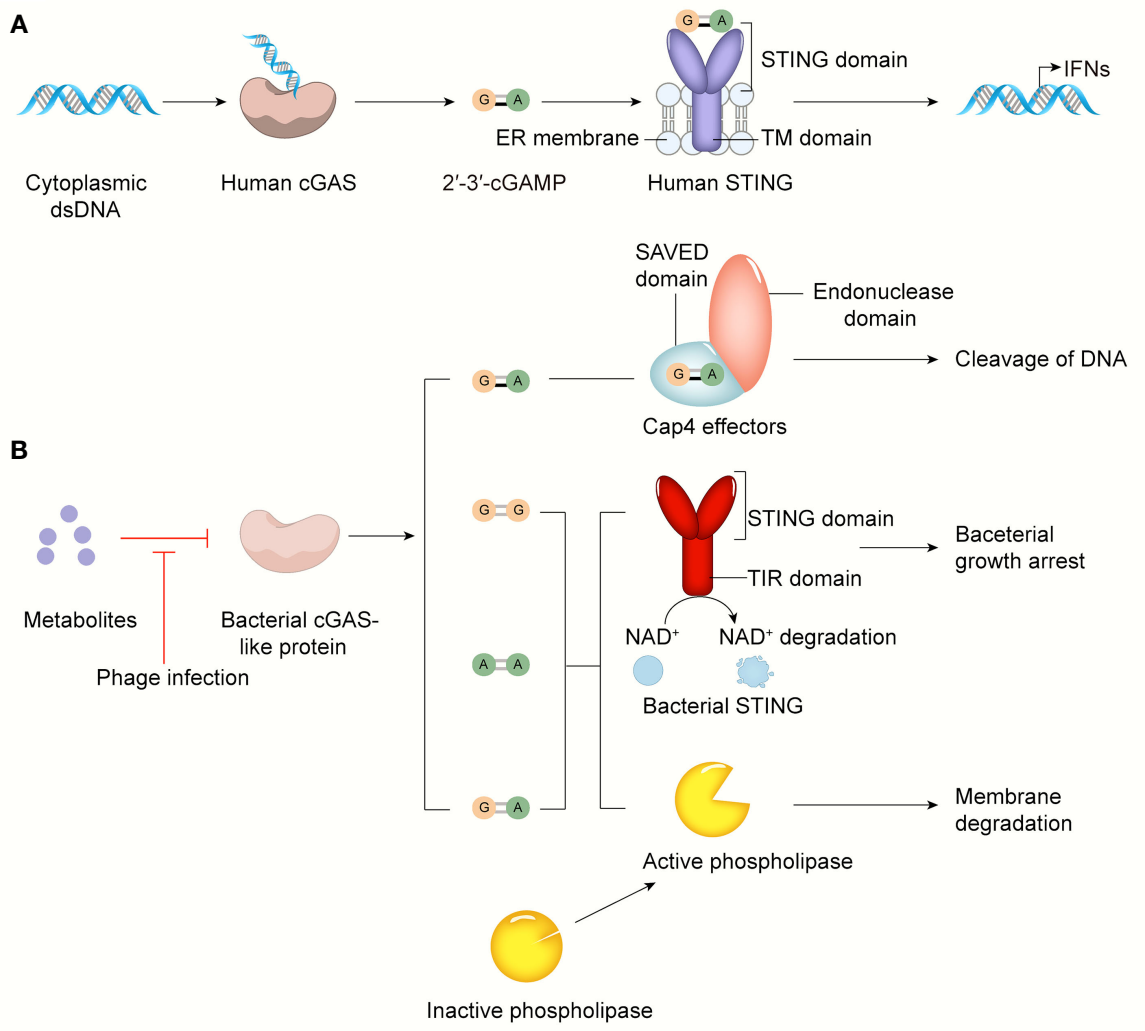
The cGAS-STING pathway in bacterial infection and bacterial immunity. **(A)** cGAS senses cytosolic dsDNA, catalyzes 2’3’-cGAMP that binds and activates STING anchored on ER, leading to IFN-I production. **(B)** The activity of the bacterial cGAS-like enzyme is inhibited by some metabolites or other unknown molecules in normal conditions. In response to phage infection, the inhibition of bacterial cGAS-like enzyme is relieved. Surprisingly, in addition to 3’3’-cGAMP, c-di-AMP, c-di-GMP, 2’3’-cGAMP can be generated by *Ab*CdnD. The SAVED domain of Cap4 recognizes 2’3’-cGAMP, then its endonuclease domain cleaves viral DNA to defend against phage infection. Bacterial STING-TIR fusion protein activated by its ligands like c-di-GMP induces bacterial growth arrest by degrading NAD^+^. Upon activated by 3’3’-cGAMP, phospholipase degrades bacterial membrane to control phage infection.

With a deeper understanding of the diversity and complexity of bacterial immune systems, researchers have speculated that these defense systems mirror those of animals. The bacterial CRISPR-Cas system, for example, is similar to the human adaptive immune system, which can form immune memories to fight specific pathogens upon re-infection (38). The cGAS-STING pathway is a crucial signaling cascade in innate immunity that regulates inflammatory diseases, autoimmune diseases, senescence, and cancer (15). Currently, most of the work regarding the relationship between the cGAS-STING signaling pathway and bacteria focuses on the controversial role of the cGAS-STING pathway during bacterial infection. Strikingly, several recent studies have reported that cGAS-like and STING-like proteins that protect bacteria against viral infection are found in bacteria, raising the possibility that the cGAS-STING pathway originates from the bacterial immune system (110, 143, 144). Next, we will review the structure and function of the cGAS-STING pathway in bacterial immunity (**Figure 4**).

## cGAS-Like Enzymes and CDNs

A bacterial four-gene operon system that contains cGAS-like and downstream effector-encoding genes and two additional genes, known as the CDN-based anti-phage signaling system (CBASS), initiates a second messenger-dependent antiviral response that is similar to that in mammalian cells (145, 146). Bacterial CD-NTases can use all four ribonucleotides to synthesize many types of CDNs and cyclic trinucleotide products. The *Vibrio cholerae* CD-NTase dinucleotide cyclase in *Vibrio* (DncV) synthesizes the second messenger 3’3’-cGAMP, *Escherichia coli* CdnE (CD-NTase in clade E) produces 3’3’ cyclic UMP-AMP, Flavobacteriaceae sp. CdnE synthesizes 3’3’ c-di-GMP, and *Enterobacter cloacae* CdnD synthesizes 3’3’3’ cyclic AMP-AMP-GMP and 3’3’-cGAMP. These bacterial CDNs or cyclic trinucleotide molecules can be distinguished from 2’3’-cGAMP, which is critical for STING activation in mammalian cells by their phosphodiester linkage specificity. The asymmetric pattern of the phosphodiester bonds in 2’3’-cGAMP has been reported in multicellular animals rather than bacteria, indicating structural changes in STING’s dominant ligand and CDN-binding pocket during evolution, which will be discussed later. However, why the phosphodiester bonds between GMP and AMP change during evolution remains unknown.

*Acinetobacter. baumannii* CdnD (*Ab*CdnD) has been unexpectedly identified to produce the noncanonical 2’-5’-linked second messenger for antiviral immunity, demonstrating that incorporation of 2’-5’ and 3’-5’ phosphodiester linkages is not a unique adaptation that evolved in eukaryotes but is also present in bacteria to subvert viral resistance (143, 147–149). CD-NTase-associated protein 4 (Cap4), a founding member of a major family of downstream bacterial receptors, can recognize various CD-NTase signals, including 2’-5’- and 3’-5’-linked bacterial nucleotide second messengers, in CBASS immunity (143). Cap4 contains a ligand-binding SAVED domain, a fusion of two CRISPR-associated Rossman fold (CARF) domains, and a promiscuous DNA endonuclease domain. After nucleotide second messenger recognition in the SAVED domain, the endonuclease domain of Cap4 is subsequently activated through ligand-induced oligomerization and induces promiscuous DNA cleavage to restrict phage replication (**Figure 4**). These SAVED CARF family proteins provide a precondition for cGAMP signaling to be a part of an antiphage defense system and suggest that the importance of linkage specificity reaches far beyond the mammalian cGAS-STING pathway.

Another unresolved question is how bacterial CD-NTase enzymes are activated to catalyze nucleotide second messenger synthesis during phage infection. To date, researchers have found that bacterial cGAS-like proteins are constitutively active *in vitro*. Moreover, DncV seems to be liberated from the inhibition of folate-like molecules only when phage infection has occurred (150). Based on the present studies, we speculate that the activity of bacterial cGAS-like proteins is normally inhibited by certain molecules, such as some metabolites, but when phages invade, these molecules are degraded or depleted under the regulation of some mechanism, thus activating the antiviral immune response. These specific molecular mechanisms need to be further explored. The bacterial antiviral response initiated by the CBASS system is independent of IFN-I, which is crucial for the mammalian antipathogen response. For example, the bacterial cGAS-like enzyme dinucleotide cyclase in *Vibrio* (DncV) synthesizes the second messenger 3’3’-cGAMP, which subsequently activates a downstream effector like phospholipase and results in the degradation of the inner bacterial membrane and cell death, thereby limiting phage replication and protecting the remaining bacterial population during phage infection (151–153) (**Figure 4**).

Intriguingly, 3’3’ c-di-GMP is considered to coordinate many different aspects of bacterial growth, behavior, and intracellular signaling, including polymer cellulose synthesis, biofilm formation, motility, virulence, and cell cycle progression (33). However, its role in the CBASS immune system, which kills bacteria or induces bacterial growth arrest, contradicts its original biological function described above. Thus, whether 3’3’ c-di-GMP, which is secreted continuously to maintain normal bacterial homeostasis, can activate CBASS downstream effector proteins is very important for bacterial survival. Researchers have conducted a series of experiments to answer this question, including analyzing the genomes of bacteria containing cGAS-STING pathways. The results showed that bacterial STING and other signaling pathways involving 3’3’ c-di-GMP rarely coexist in bacteria. In this way, bacteria can effectively avoid potentially catastrophic conflicts caused by the dual effects of 3’3’ c-di-GMP.

## STING-Like Protein

Many structurally divergent effector domains can be activated by CD-NTase products to fight against phage infection, including proteases, phosphodiesterases, and potentially pore-forming transmembrane proteins (145, 154). Among them, some proteins are found to contain a C-terminal STING domain, further suggesting that the intact cGAS-STING pathway may be preserved from bacteria to humans. X-ray crystallography analysis showed that the crystal structures of proteins in *Flavobacteriaceae* sp. and *Capnocytophaga granulosa* exhibited obvious homology to human STING. Bacterial STING is encoded within bacterial defense islands and has been newly identified as a prokaryotic member of the STING family of functional cyclic dinucleotide receptors (144).

The overall architecture of bacterial STING is similar to that of mammalian STING, and both adopt a canonical V-shaped, homodimeric architecture with a hydrophobic α-helix stem. However, the bacterial STING protein is 20% smaller and more compact than mammalian STING, mainly due to a lack of extension in the β-strand lid domain, a terminal α-helix, and an unstructured C-terminal tail (51, 66, 70, 72). These mammalian-specific insertions allowed STING to evolve to induce autophagy and regulate innate immune responses dependent on IFN-I. This structural difference partly explains why the bacterial CBASS immune system takes a suicidal approach rather than relying on the IFN-I response to fight against phage infection.

In addition, there are differences in the amino acid residues of the CDN-binding pockets of bacterial STING and human STING (144), indicating that they may have different ligand preferences. Unlike human STING, which binds CDNs with no sequence-specific contact, a conserved residue in the CDN-binding pocket of bacterial STING makes sequence-specific contacts to the guanosine nucleobase of c-di-GMP, which is consistent with its high-affinity recognition of c-di-GMP. Furthermore, bacterial STING exhibits a weaker affinity for 3’3’-cGAMP, and it cannot recognize mammalian 2’3’-cGAMP since there is not enough space in its CDN-binding pocket to accommodate the free 3’-OH of 2’3’-cGAMP due to the presence of another conserved residue in bacterial STING; in contrast, human STING recognizes 2’3’-cGAMP with high affinity by making an additional contact between the human STING residue and the phosphodiester backbone (155).

Bacterial STING exists as a fusion protein appended to the Toll/interleukin-1 receptor (TIR) adaptor domain or predicted transmembrane (TM) effector modules, and the STING-TIR fusion protein is the most dominant form of STING. The TLR domain has been widely recognized to play an important role in protein-protein interactions to defend against various pathogens in mammals. Some TIR domains can degrade β-nicotinamide adenine dinucleotide (NAD^+^), required for cellular metabolism (156, 157).

After recognizing c-di-GMP, bacterial STING assembles into long-ordered filaments with fourfold symmetry. Filaments contain parallel-stacked STING homodimers arranged in an orderly manner, consistent with the activation mechanism of human STING discussed above (51, 71). In contrast to c-di-GMP, the weak agonist 3’3’-cGAMP induced only partial formation of filaments, suggesting that c-di-GMP plays a crucial role in the process by which bacterial STING oligomerizes into filaments. Mutations in the CDN-binding domain of bacterial STING blocks the formation of filaments without affecting the binding of c-di-GMP and leads to the loss of STING NADase activity, confirming that filament formation controls the activation of STING, as well as the rapid cleavage of NAD^+^ mediated by the TIR domain (144). NAD^+^ destruction that occurs after the activation of STING-TIR fusion proteins results in cell death to halt phage propagation, which is different from the immune response activated by human STING, which relies on the expression of antipathogen-related genes and proteins (158) (**Figure 4**).

STING-TIR fusion proteins also exist in some invertebrates, such as *Crassostrea gigas*. However, STING-TIR fusion proteins in *C.gigas* can bind tightly to 2’3’-cGAMP (144). In addition, the residues of STING CTT differ across species, resulting in different activation preferences for downstream pathways, including IFN-I response and NF-κB response. These findings indicate that the structure, composition, and function of STING are all changing under selective pressure, but the reasons for these changes are still unknown.

In summary, bacterial CBASS immunity uses the CD-NTase enzyme, a hcGAS homolog, to catalyze the production of diverse nucleotide second messengers, recognized by the bacterial STING-TIR fusion protein to initiate an antiviral response by driving the destruction of NAD^+^. Although there are many differences in the cGAS-STING pathways of mammals and bacteria, each of the fundamental components that define the human cGAS-STING pathway is functionally and structurally shared within ancient bacterial CBASS immunity. The preserved antipathogen defense from bacteria to humans provides a deeper understanding of how the modern cGAS-STING pathway has been shaped during evolution, providing directions for developing therapeutics for cGAS-STING-related diseases.

## Discussion

In the last decade, a series of studies in cytosolic surveillance systems have made fruitful progress and demonstrated a critical role of the cGAS-STING signaling pathway in bacterial infection. Both extracellular and intracellular bacteria activate cGAS-STING signaling in host cells through PAMPs, including bacterial DNA, CDNs, and LPS, or DAMPs, such as cytosolic DNA released from host mitochondria and the nucleus. Structural information about the main molecules of cGAS-STING signaling has uncovered their regulatory mechanisms, ligand-binding sites, conformational changes, intracellular localization, and function in signal propagation. Moreover, unlike its protective effect against viruses, the activity of the cGAS-STING signaling pathway in bacterial infection may not always be protective. In addition to the induction of cell apoptosis by the IFN-I response during *F. novicida* and *L.monocytogenes* infection, activation of the cGAS-STING pathway can promote bacterial replication during *B. abortus* infection and intracellular bacterial survival during *S. aureus* infection. The cGAS-STING pathway can also affect the outcomes of bacterial infections by participating in the mediation of some important physiological or pathological processes such as blood coagulation and autophagy. The cGAS-STING pathway has also been found in bacteria to defend against phage infection, which provides evolutionary insights into the development of mammalian cGAS-STING signaling and the ongoing host-bacterial arms race.

In addition to the cytoplasm and membrane, hcGAS is also found in the nucleus, easily contact genomic DNA. Structural analysis showed that cGAS is anchored to the nucleosome acidic patch of histone H2A-H2B. Binding to the nucleosome blocks the interaction of cGAS with dsDNA by occupying the strong dsDNA binding surface on cGAS and preventing cGAS dimerization, contributing to the inhibition of cGAS activity. The self-nonself discrimination of host nuclear dsDNA by cGAS can protect the host from autoimmune attack (49, 56, 159). Bacterial cGAS-like enzymes also have easy access to dsDNA, exposed in the cytoplasm without a nuclear membrane. Similarly, the activity of bacterial cGAS-like enzymes is suppressed under normal conditions to avoid the induction of bacterial death by aberrant cGAS activation. The differences and similarities in the mechanism of cGAS inhibition between bacteria and humans remain to be studied.

Despite the current progress, many questions remain to be answered in the future. For instance, can STING mediate physiological or pathological processes other than innate immune responses, such as cell metabolism, independent of cGAS or TBK1, and how are these functions finely orchestrated to maintain cellular homeostasis?

Are there any rules determining whether the cGAS-STING signaling pathway has protective or detrimental effects in various bacterial infections? Does cGAS-STING play different roles in different diseases caused by the same bacteria? Precisely how is STING transported from the ER to the Golgi complex? What is the significance of certain small changes that occurred in the cGAS-STING pathway during evolution? With the resolution of these questions in the future, we expect to effectively control bacterial infection and protect the host from injury by fine-tuning the cGAS-STING signaling pathway.

## Author Contributions

NL and XP have contributed equally to this work. PJ and HZ are corresponding authors. All authors contributed to the article and approved the submitted version.

## Conflict of Interest

The authors declare that the research was conducted in the absence of any commercial or financial relationships that could be construed as a potential conflict of interest.

## Publisher’s Note

All claims expressed in this article are solely those of the authors and do not necessarily represent those of their affiliated organizations, or those of the publisher, the editors and the reviewers. Any product that may be evaluated in this article, or claim that may be made by its manufacturer, is not guaranteed or endorsed by the publisher.
